# Treatment Patterns of New Users of Fluticasone Furoate/Vilanterol in Asthma and COPD in UK Primary Care: Retrospective Cohort Study

**DOI:** 10.1007/s41030-019-0092-z

**Published:** 2019-04-24

**Authors:** Daniel Dedman, Sonia J. Coton, Rebecca E. Ghosh, Wilhelmine Meeraus, Courtney Crim, Catherine Harvey, Justyna Amelio, Sarah H. Landis

**Affiliations:** 1 Clinical Practice Research Datalink (CPRD), Medicines and Healthcare Products Regulatory Agency (MHRA), London, UK; 2 GlaxoSmithKline plc, Epidemiology, Value Evidence and Outcomes, Stockley Park, Uxbridge, UK; 3 GlaxoSmithKline plc, Research and Development, Research Triangle Park, NC USA; 4 GlaxoSmithKline plc, Global Clinical Safety and Pharmacovigilance, Stockley Park, Uxbridge, UK; 5 GlaxoSmithKline plc, Epidemiology, Value Evidence and Outcomes, Stevenage, UK

**Keywords:** Asthma, Chronic obstructive pulmonary disease (COPD), Fluticasone furoate/vilanterol (FF/VI), Primary care

## Abstract

**Introduction:**

This retrospective database study explored treatment patterns and potential off-label prescribing among patients newly prescribed fluticasone furoate/vilanterol (FF/VI) in a UK primary care setting.

**Methods:**

In Europe, FF/VI is approved in two strengths: 100/25 µg for adults with chronic obstructive pulmonary disease (COPD) and 100/25 µg or 200/25 µg for treatment of asthma in patients aged 12 or older. Using electronic health records from the Clinical Practice Research Datalink, new users of FF/VI or other inhaled corticosteroid/long-acting beta-agonist fixed-dose combination products were identified and classified into one of three groups: COPD diagnosis, asthma diagnosis, and other diagnosis (not COPD or asthma).

**Results:**

During 2014–2015, 4373 patients initiated FF/VI: 3380 on FF/VI 100/25 (65% in the COPD diagnosis group) and 993 on FF/VI 200/25 (51% in the asthma diagnosis group). During up to 12 months of follow-up, the median number (interquartile range) of prescriptions of the index strength issued per patient was 7 (2–8) for FF/VI 100/25 and 5 (2–8) for FF/VI 200/25; most new users did not change from the index strength prescribed (93.0% COPD; 89.7% asthma, of all patients initiating treatment with FF/VI). Potential off-label FF/VI prescribing in children < 12 years old was rare (< 0.29% in the combined asthma and other diagnosis groups), and up to one in five new users of FF/VI with COPD were potentially prescribed FF/VI 200/25 off-label during the study period. Much of the potential off-label prescribing in COPD occurred in patients with a history of asthma, those presenting with greater disease severity, and/or prior treatment with high-dose steroids.

**Conclusions:**

The prescription of FF/VI is rare in children under 12 years of age in the UK, according to our findings, but up to one in five COPD patients in the UK may have been prescribed FF/VI 200/25, some of which may have been off-label.

**Funding:**

This study was funded by GlaxoSmithKline plc (study 205052).

**Study Registration:**

GlaxoSmithKline plc Clinical Trial Registry study number 205052.

**Electronic supplementary material:**

The online version of this article (10.1007/s41030-019-0092-z) contains supplementary material, which is available to authorized users.

## Introduction

Inhaled corticosteroids (ICS) and long-acting beta-agonists (LABAs) are mainstays of treatment for chronic obstructive pulmonary disease (COPD) and asthma, both of which are common respiratory diseases globally [1, 2]. There is strong evidence that combining an ICS with a LABA is more effective at reducing exacerbations and improving lung function and health status in patients with stable COPD than each component individually [3–7]. In asthma, the addition of a LABA to an ICS improves lung function and symptom scores while reducing the number of asthma exacerbations and nocturnal asthma symptoms [8], and is thus preferred to increasing the ICS dose to achieve asthma control [2].

Fluticasone furoate/vilanterol (FF/VI) is a fixed-dose combination (FDC) ICS/LABA for once-daily inhalation that is available in two dose strengths, 100/25 µg and 200/25 µg. In Europe, the 100/25 µg strength is indicated for the symptomatic treatment of adults with COPD who have a history of exacerbations despite bronchodilator therapy. Both strengths (100/25 µg and 200/25 µg) are approved for regular treatment of patients with asthma aged ≥ 12 years whose symptoms are not controlled with an ICS plus an inhaled short-acting β_2_ agonist, or whose symptoms are adequately controlled with an ICS/LABA [9].

It is important to study how newly marketed drugs are prescribed in clinical practice following their launch, especially regarding possible off-label prescribing. While prescribers may be familiar with the class of medication, they may lack knowledge as to the correct use of the newly marketed product and assume it has a similar indication to other medications in the class, or they may extrapolate clinical trial data to populations that were not investigated. In the United Kingdom (UK), prescribed medication is commonly accessed via general practitioners (GPs) and is recorded in primary care electronic health records (EHR) maintained in general practice. These records also include interactions with secondary care. This observational study using EHR from the Clinical Practice Research Datalink (CPRD) was conducted to investigate treatment patterns among new users of FF/VI in the UK primary care setting, including potential indication for use and prescribing of concomitant medication. Two aspects of potential off-label prescribing were explored: FF/VI (any strength) in children aged < 12 years, and FF/VI 200/25 in patients with evidence of a COPD diagnosis.

## Methods

### Study Design

This retrospective, longitudinal, observational study utilised primary care EHR from CPRD, which holds data that are broadly representative in terms of age, sex, and ethnicity of the UK population [10]. FF/VI was first marketed in the UK in January 2014. The study population included new users of FF/VI or another ICS/LABA FDC (fluticasone propionate/salmeterol xinafoate, budesonide/formoterol, beclometasone/formoterol, and fluticasone propionate/formoterol) during the 2-year inclusion period of 1 January 2014 to 31 December 2015 (the date of the first prescription was the index date). New prescribing was defined for a specific FDC product as never having had a prescription for FF/VI or another index ICS/LABA FDC recorded previously (prior prescribing of a different ICS/LABA FDC and concomitant prescribing of other respiratory medications on the index date were permitted). A patient could contribute information on more than one index medication if they met the “new user” definition for multiple medications during the inclusion period. Patients were required to have at least 12 months of data prior to the index date to allow the characterisation of disease status, demographics, and clinical characteristics. Patients were followed from their index date until the first of the following events: (1) 12 months post index date, (2) death (censored), or (3) leaving their GP practice (censored), potentially allowing up to 12 months of follow-up time. The other ICS/LABA FDC cohort provided a benchmark for patient characteristics of new users of FF/VI, but was not included as a formal comparison group (no statistical comparisons were made).

New users were classified sequentially into three mutually exclusive diagnosis groups using validated coding lists and algorithms [11, 12]. First, a COPD diagnosis group was defined as new users with a COPD diagnosis recorded any time up to the end of their follow-up and aged ≥ 35 years at the time of their first ever recorded COPD diagnosis. Patients in the COPD diagnosis group were further stratified by ‘asthma history’ (i.e. an asthma diagnosis recorded prior to and including the index date). Next, the asthma diagnosis group was defined as those patients who did not meet the criteria for COPD and had an asthma diagnosis recorded at any time up to the end of their follow-up. The other diagnosis group was defined as patients who did not meet the criteria for either the COPD or asthma groups.

#### Demographics of New Users of FF/VI or Another ICS/LABA FDC

Patient characteristics (gender, age, smoking status, and body mass index) at the index date and the prescribing of other asthma or COPD medications in the 12 months prior to the index date were described; for the ICS category, the highest dose prescribed (high/medium/low) was recorded based on Global Initiative for Asthma conversion guidelines [13]. Disease burden variables for the COPD group were described in the 12 months prior to the index date (unless otherwise noted): (1) forced expiratory volume in the first second (FEV_1_) as a percentage of that predicted, and FEV_1_/forced vital capacity (FVC) ratio based on the most recent lung function test in the 24 months prior to the index date (note: the primary care record does not always record pre- or postbronchodilator status); (2) Medical Research Council (MRC) dyspnoea score; and (3) acute exacerbations of COPD using a validated algorithm [14]. For the primary analysis of acute exacerbations of COPD, we captured episodes recorded in the GP database alone; a sensitivity analysis of a subset of patients whose GP-recorded data could be linked to the Hospital Episode Statistics (HES) was conducted to more fully capture exacerbation episodes that required hospitalisation (see the Electronic supplementary material, ESM, for more detail). Exacerbation episodes were defined as COPD-specific treatments with antibiotics combined with oral corticosteroid (OCS) and/or medical diagnosis codes for COPD exacerbations or acute bronchitis, or as a hospitalisation for COPD. For the asthma diagnosis group, asthma exacerbations in the 12 months prior to the index date were defined as a prescription for an OCS within ± 14 days of an asthma code or an asthma hospital referral or asthma exacerbation events requiring hospitalisation. Asthma exacerbation episodes were reported in the main analysis using only the GP-recorded data, and in the sensitivity analysis for patients whose data could be linked to HES data.

#### FF/VI Prescribing Patterns

For new users of FF/VI 100/25 and FF/VI 200/25, the total count and median [interquartile range (IQR)] number of FF/VI prescriptions over up to 12 months of follow-up was calculated. Among patients who received at least two prescriptions of FF/VI (any strength), the proportion of new users with an escalation or reduction in FF/VI strength during the follow-up was reported by diagnosis group. Concomitant prescribing of other respiratory therapies at the index date was also identified, with concomitant therapy defined as at least two continuous prescriptions for the other respiratory therapy that started either before or up to 30 days after the index date and overlapped for at least 30 days with the index FF/VI treatment.

#### Potential Off-Label Prescribing of FF/VI

Potential off-label prescribing of FF/VI (any strength) in children aged < 12 years was calculated as the number of patients aged < 12 years old who received FF/VI (any strength) divided by all patients in the asthma and other diagnosis groups (by definition, all patients in the COPD diagnosis group were aged ≥ 35 years and therefore excluded from the denominator population).

Potential off-label prescribing of FF/VI 200/25 in patients with evidence of COPD was considered using two alternative approaches that accounted for COPD patients with and without an ‘asthma history’, as it is not possible to determine from a retrospective database whether FF/VI 200/25 was prescribed to treat COPD or asthma. The first approach provides upper bound estimates, assuming that all COPD patients with an asthma history receive FF/VI 200/25 for treatment of their COPD either (1) at the index date (number of patients with COPD prescribed FF/VI 200/25 *as their index prescription* divided by all patients in the COPD diagnosis group), or (2) at any time during follow-up (number of patients with COPD prescribed FF/VI 200/25 *at any time during follow*-*up* divided by all patients in the COPD diagnosis group). The second approach provides lower-bound estimates by assuming that COPD patients with an asthma history are prescribed FF/VI 200/25 for treatment of their asthma. For this approach, the proportion was calculated: (1) at the index date (number of patients with COPD and no asthma history prescribed FF/VI 200/25 *as their index prescription* divided by all patients in the COPD diagnosis group), or (2) at any time during follow-up (number of patients with COPD without asthma history prescribed FF/VI 200/25 *at any time during follow*-*up* divided by all patients in the COPD diagnosis group).

### Statistical Analysis

Patient characteristics were described using mean (standard deviation) and median (IQR) for quantitative variables and numbers (*n*), and proportions (%) for categorical variables. COPD and asthma exacerbations during the 12 months prior to the index date were described as 0, 1, 2+ events and were expressed as rates per person-year with the corresponding 95% confidence interval (CI). When data were missing, the number of patients with missing data was reported, but these patients were excluded from the summary statistics.

### Ethics

The CPRD contains only de-identified patient data. The CPRD has broad Health Research Authority National Research Ethics Service Committee ethics and governance approval for purely observational research using the primary care data and established data linkages. The study protocol (16_229R) was approved by the CPRD Independent Scientific Advisory Committee. Tables were adapted to suppress patient counts of less than five, in compliance with CPRD policy on managing anonymisation and the risk of identification in observational research.

## Results

**Fig. 1:**
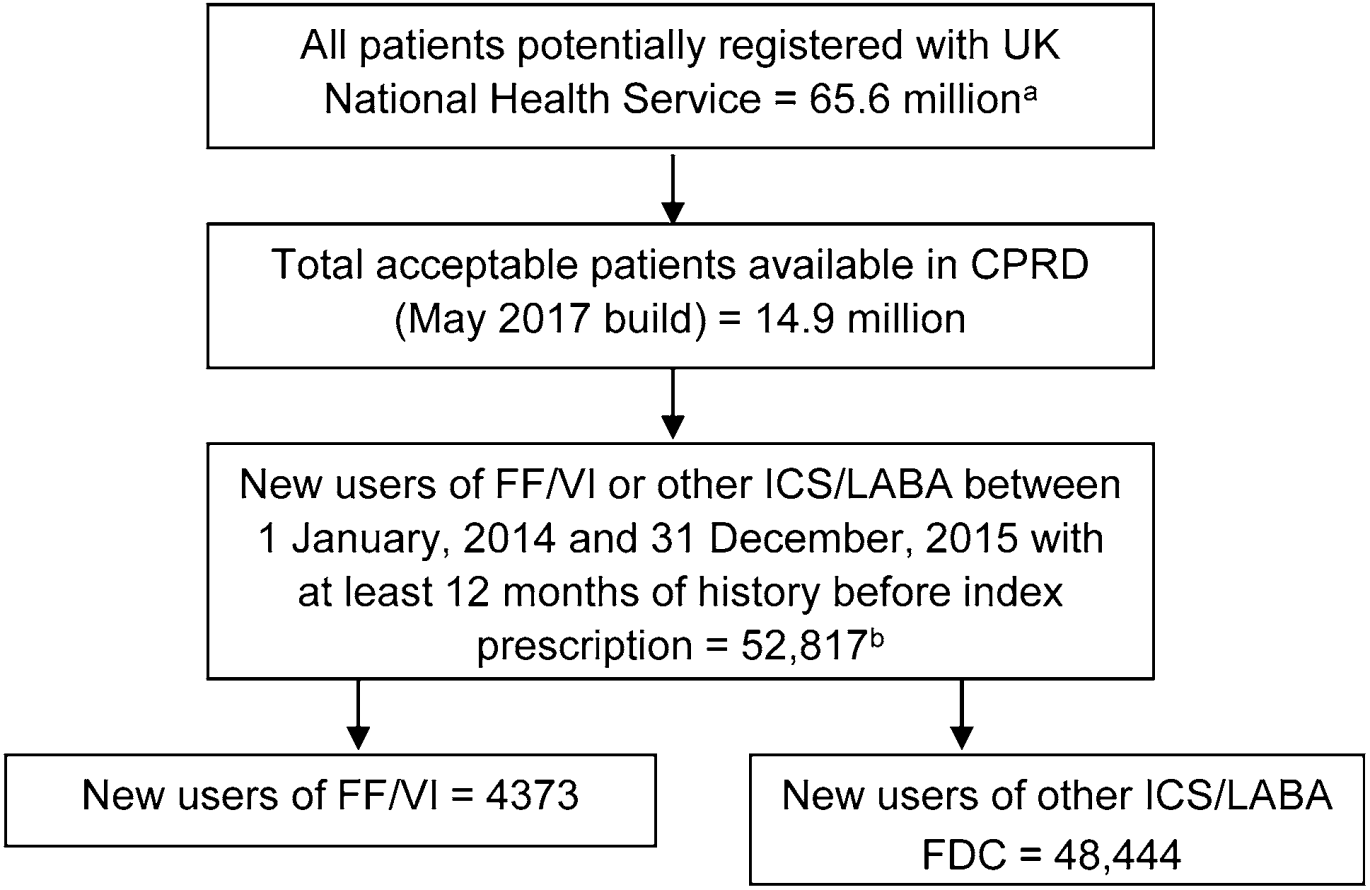
CONSORT diagram.* Superscript letter a* indicates the mid-year estimate of the UK population for 2016 from the Office for National Statistics.* Superscript letter b* indicates that patients contributed information on more than one treatment if they met the “new user” definition for more than one medication during the inclusion period. *CPRD* Clinical Practice Research Datalink, *FDC* fixed-dose combination, *FF/VI* fluticasone furoate/vilanterol, *ICS/LABA* inhaled corticosteroid/long-acting beta agonist

New users of FF/VI were classified into one of three groups: COPD diagnosis (further stratified by asthma history), asthma diagnosis, and other diagnosis (not COPD or asthma). During the study period, 4373 patients initiated FF/VI: 3380 on FF/VI 100/25 (29% COPD with an asthma history, 36% COPD without an asthma history, 31% asthma, 4% other) and 993 on FF/VI 200/25 (25% COPD with an asthma history, 20% COPD without an asthma history, 51% asthma, 4% other) (Fig. 1, Table 1). During the same time period, 48,444 patients initiated other ICS/LABA FDCs, with just over half categorised as asthma patients (14% COPD with an asthma history, 18% COPD without an asthma history, 57% asthma, 11% other). The most common diagnostic codes in the other diagnosis group were related to upper or lower respiratory tract infections. Following initiation, about three-quarters of the patients contributed data for the full 12-month follow-up period; a patient leaving their GP practice was the most common reason for censoring.

**Table 1:** Demographic characteristics at baseline for the COPD diagnosis group, asthma diagnosis group, and other diagnosis group by index medication

	Patients in the COPD diagnosis group (*n* = 16,629)^a^						Patients in the asthma diagnosis group (*n* = 27,051)^a^						Patients in the other diagnosis group (*n* = 5250)^a^					
	FF/VI 100/25		FF/VI 200/25		Other ICS/LABA		FF/VI 100/25, *n* = 1052		FF/VI 200/25		Other ICS/LABA		FF/VI 100/25, *n* = 123		FF/VI 200/25, *n* = 43		Other ICS/LABA, *n *= 5295	
	*N* = 2205		*N* = 448		*N* = 15,576				*N* = 502		*N* = 27,573							
	No.	(%)^b^	No.	(%)^b^	No.	(%)^b^	No.	(%)^b^	No.	(%)^b^	No.	(%)^b^	No.	(%)^b^	No.	(%)^b^	No.	(%)^b^
Age at index date																		
Mean (SD)	69.4	10.4	68.1	11.1	69.0	11.2	49.2	19.5	53.1	18.0	47.0	20.5	63.8	17.3	63.2	17.2	58.6	19.6
Gender																		
Female	1105	50.1	209	46.7	7855	50.4	681	64.7	334	66.5	16,876	61.2	52	42.3	15	34.9	2324	43.9
Smoking status																		
Current smoker	841	38.2	161	35.9	6131	39.4	255	24.3	89	17.8	5461	20.5	43	36.1	6	14.3	1176	23.3
Ex-smoker	1200	54.5	231	51.6	7659	49.3	278	26.5	145	28.9	6480	24.3	39	32.8	18	42.9	1561	30.9
Never smoked	163	7.4	56	12.5	1758	11.3	515	49.1	267	53.3	14,701	55.9	37	31.1	18	42.9	2321	45.9
*Missing*^c^	*1*	*0.1*	*0*	*0.0*	*28*	*0.9*	*4*	*0.4*	*1*	*0.2*	*931*	*3.4*	*4*	*3.3*	*1*	*2.3*	*237*	*4.5*
Body mass index (kg/m^2^)																		
Mean (SD)	27.9	6.3	28.3	6.9	27.7	6.5	30.1	7.0	31.1	6.9	29.5	6.8	29.0	6.3	29.8	7.9	29.1	6.9
*Missing*^c^	*86*	*3.9*	*25*	*5.6*	*1158*	*7.4*	*4*	*0.4*	*1*	*0.2*	*931*	*3.4*	*43*	*36.1*	*6*	*14.3*	*1176*	*23.3*
Mean (SD) follow-up time (days)	328 (86)		322 (92)		319 (95)		323 (88)		333 (82)		328 (86)		284 (123)		322 (92)		310 (104)	

Italics were used to distinguish the missing values from the non missing values

*COPD* chronic obstructive pulmonary disease, *FF/VI* fluticasone furoate/vilanterol, *ICS/LABA* inhaled corticosteroid/long-acting beta agonist, *SD* standard deviation

^a^Patients can qualify for more than one qualifying index medication, which is reflected in the higher number of records when summing medication categories within an exposure cohort

^b^Unless otherwise specified

^c^Percentages were calculated separately for those with missing and without missing data

### Demographic Characteristics of New Users of FF/VI or Another ICS/LABA FDC

Demographic characteristics are presented separately for the COPD, asthma, and other diagnosis groups (Table 1, Table S1 in the ESM). The mean age of new users of FF/VI or another ICS/LABA FDC in the COPD diagnosis group was 68.1–69.4 years, and was marginally higher in the COPD without an asthma history diagnosis group (Table S1 in the ESM). The mean age of the asthma diagnosis group showed more variation over the three exposure cohorts (i.e. FF/VI 100/25, FF/VI 200/25, and other ICS/LABA FDC), ranging from 47.0 years for other ICS/LABA FDC to 53.1 years for FF/VI 200/25.

Females comprised about half of new users in the COPD diagnosis group over the three exposure cohorts (range 46.7–50.4%), while the proportion of females was greater in the asthma group (range 61.2%–66.5%). The proportion of current smokers was highest in the COPD diagnosis group without an asthma history (range 39.9–43.4%), followed by COPD patients with an asthma history (range 32.8–35.7%), and was lowest among patients in the asthma diagnosis group (range 17.8–24.3% (Table 1, Table S1 in the ESM).

### Prior and Concomitant Respiratory Medication Prescribing Among New Users of FF/VI or Another ICS/LABA FDC

In the 12 months prior to the index date, FF/VI users were more likely to have received a different ICS/LABA medication (69.1% for FF/VI 100/25 and 67.9% for FF/VI 200/25) than patients initiating another ICS/LABA FDC (42.0%); past prescribing of long-acting muscarinic antagonist (LAMA) monotherapy and LABA/LAMA dual therapy was also greater for FF/VI users (Fig. S1 in the ESM). A large proportion of the COPD patients had been prescribed a high-dose ICS (alone or in combination with LABA) in the 12 months prior to initiating FF/VI 200/25 (37.9% in COPD patients without an asthma history’ and 57.2% in COPD patients with an asthma history). These proportions were similar in patients initiating FF/VI 100/25. Chronic use of OCS (≥ 4 prescriptions, with a maximum gap between two prescriptions equal to 30 days) was present in fewer than 10% of patients for both FF/VI strengths. Among patients in the asthma diagnosis group, nearly all were prescribed a short-acting bronchodilator (SABD) in the 12 months prior to the index date, and this was similar for FF/VI (any strength) and another LABA/ICS FDC. Past prescribing of another ICS/LABA was more common for the FF/VI group, especially the group initiating FF/VI 200/25, than for the LABA/ICS FDC group, whereas ICS monotherapy prescribing was highest for the LABA/ICS FDC group (Fig. S2 in the ESM).

Among patients in the COPD diagnosis group, LAMA was the most commonly prescribed concomitant maintenance medication (i.e. multiple inhaler triple therapy) at the index date (59.7% for FF/VI 100/25 and 51.8% for FF/VI 200/25); concomitant LAMA prescribing was less frequent in the LABA/ICS FDC group (45.4%). In the asthma diagnosis group, SABD concomitant prescribing was also the most common (63.3% for FF/VI 100 and 58.6% for FF/VI 200), followed by leukotriene receptor antagonists (17.9% for FF/VI 200/25; 9.5% for FF/VI 100/25; 8.6% for other LABA/ICS FDC).

### COPD and Asthma Disease Severity Among New Users of FF/VI or Another ICS/LABA FDC

Among patients in the COPD group, new users of FF/VI 200/25 had the highest rates of COPD exacerbation in the year prior to index date (1.53 per person-year, 95% CI 1.42, 1.65) compared with new users of FF/VI 100/25 (1.36 per person-year, 95% CI 1.31, 1.41) and new users of another ICS/LABA FDC (1.13 per person-year, 95% CI 1.12, 1.15; Table 2). The HES-linked subsample, which facilitated the more complete capture of exacerbation episodes treated in secondary care, had slightly higher rates but followed the same pattern as the full CPRD sample (Table S2 in the ESM). Among this subset of patients, the rate of moderate COPD exacerbations was 1.53 per person per year (95% CI: 1.37, 1.72) for new users of FF/VI 200/25, 1.37 per person per year (95% CI: 1.30, 1.45) for new users of FF/VI 100/25, and 1.18 per person per year (95% CI: 1.15, 1.20) for new users of another ICS/LABA FDC. Airflow limitation as measured by FEV_1_ percent predicted was similar among the three drug exposure cohorts (Table 2), with 7–8% of patients with very severe, grade 4 (FEV_1_ < 30%) airflow limitation. Breathlessness as measured by MRC dyspnoea score was more severe in COPD patients initiating FF/VI (100/25 or 200/25) than in COPD patients initiating another ICS/LABA FDC.

**Table 2:** Disease severity at baseline for the COPD diagnosis group and asthma diagnosis group by index medication

Patients in COPD diagnosis group						
	FF/VI 100/25, *n* = 2205		FF/VI 200/25, *n* = 448		Other ICS/LABA FDC, *n* = 15,576	
	No.	(%)^a^	No.	(%)^a^	No.	(%)^a^
COPD exacerbations at baseline (recorded in primary care only)						
Rate per person year (95% CI)	1.36	(1.31, 1.41)	1.53	(1.42, 1.65)	1.13	(1.12, 1.15)
0 events	847	38.4	152	33.9	6836	43.9
1 event	554	25.1	120	26.8	4133	26.5
≥ 2 events	804	36.5	176	39.3	4607	29.6
FEV_1_ percent predicted at baseline						
Mean (SD)	55.59	19.47	56.09	18.87	56.79	19.15
Mild, grade 1 (≥ 80%)	198	11.0	38	11.0	1391	11.5
Moderate, grade 2 (≥ 50% to < 80%)	861	48.0	174	50.6	6071	50.3
Severe, grade 3 (≥ 30% to < 50%)	581	32.4	108	31.4	3750	31.0
Very severe, grade 4 (< 30%)	155	8.6	24	7.0	866	7.2
*Missing*^b^	*410*	*18.6*	*104*	*23.2*	*3498*	*22.5*
FEV_1_/FVC ratio at baseline						
Mean (SD)	59.01	18.32	59.42	17.61	60.85	16.00
< 70%	1286	79.8	237	75.5	7832	73.4
*Missing*^b^	*593*	*26.9*	*134*	*29.9*	*4900*	*31.5*
Dyspnoea at baseline (MRC grade)						
Mean (SD)	2.85	0.98	2.84	1.06	2.72	1.00
Grade 1	116	6.4	32	9.5	1017	9.4
Grade 2	582	32.4	104	31.0	3929	36.2
Grade 3	622	34.6	105	31.2	3430	31.6
Grade 4	403	22.4	76	22.6	2055	18.9
Grade 5	75	4.2	19	5.7	419	3.9
*Missing*^b^	*407*	*18.5*	*112*	*25.0*	*4726*	*30.3*

Patients in asthma diagnosis group						
	FF/VI 100/25, *n* = 1052		FF/VI 200/25, *n* = 502		Other ICS/LABA FDC, *n* = 15,576	
	No.	(%)^a^	No.	(%)^a^	No.	(%)^a^
Asthma exacerbations at baseline (recorded in primary care only)						
Rate per person year (95% CI)	0.09	(0.07, 0.11)	0.20	(0.17, 0.25)	0.08	(0.08, 0.08)
0 events	967	91.9	420	83.7	25,593	92.8
1 event	76	7.2	64	12.7	1795	6.5
≥ 2 events	9	0.9	18	3.6	185	0.7

Italics were used to distinguish the missing values from the non missing values

*CI* confidence interval, *COPD* chronic obstructive pulmonary disease, *FDC* fixed-dose combination, *FEV*_*1*_ forced expiratory volume in 1 s, *FF/VI* fluticasone furoate/vilanterol, *FVC* forced vital capacity, *ICS/LABA* inhaled corticosteroid/long-acting beta-agonist, *MRC* Medical Research Council, *SD* standard deviation

^a^Unless otherwise specified

^b^Percentages were calculated separately for those with missing and without missing data

Rates of exacerbations in the asthma diagnosis group in the year prior to index date were also highest in new users of FF/VI 200/25 (0.20 per person-year, 95% CI 0.17, 0.25) compared with FF/VI 100/25 (0.09 per person-year, 95% CI 0.07, 0.11), and ICS/LABA FDC (0.08 per person-year, 95% CI 0.08, 0.08; Table 2). In the HES-linked subsample, the exacerbation rates were slightly higher but followed the same pattern as for the full CPRD sample (Table S2 in the ESM).

### Exposure to FF/VI or Another LABA/ICS FDC

The median number of prescriptions of the index strength issued per patient during up to 12 months of follow-up was 7 (IQR 2–8) for FF/VI 100/25 and 5 (IQR 2–8) for FF/VI 200/25. Between a fifth and a quarter of new users had only one recorded prescription of their index FF/VI strength during the study. Adherence to treatment was assessed for patients with the full 12 months of follow-up data, corresponding to approximately two-thirds of the COPD patients and one-third of the asthma patients (Table S3 in the ESM). In COPD, 62.0% and 46.8% of patients had a medication possession ratio and proportion of days covered by prescribed treatment, respectively, of at least 80%. In asthma, 50.9% and 37.7% of patients had a medication possession ratio and proportion of days covered by prescribed treatment, respectively, of at least 80%.

Most new users did not change from the index strength prescribed over the subsequent 12 months (93.0% COPD; 89.7% asthma, of all patients initiating treatment with FF/VI). Among COPD patients, those initiating on FF/VI 200/25 were more likely to receive just one prescription of this strength, compared with those initiating on FF/VI 100/25 (26.1% vs. 13.7%, respectively). For asthma patients, the proportion of FF/VI 100/25 and FF/VI 200/25 initiators with just one prescription of the index strength was similar (22.6% and 21.5%, respectively).

**Fig. 2:**
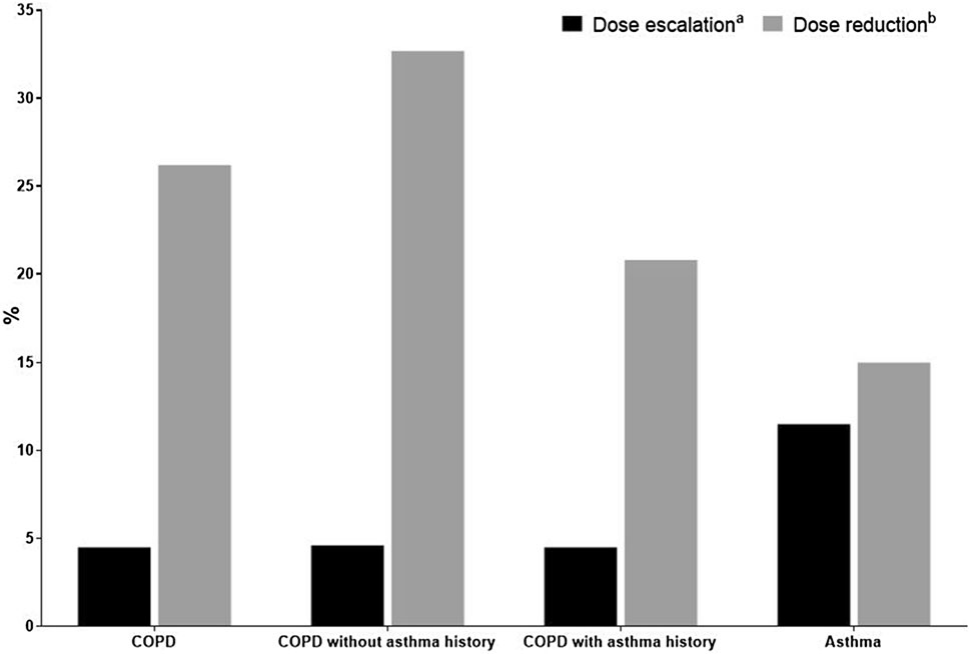
Dose adjustment according to diagnosis group (among patients who received ≥ 2 prescriptions of FF/VI).* Superscript letter a *indicates that the denominator includes new users of FF/VI 100/25 with at least two prescriptions of FF/VI (COPD = 1920, COPD without an asthma history = 1050, COPD with an asthma history = 870, asthma = 853). *Superscript letter b *indicates that the denominator includes new users of FF/VI 200/25 with at least two prescriptions of FF/VI (COPD = 378, COPD without an asthma history = 171, COPD with an asthma history = 207, asthma = 414). *COPD* chronic obstructive pulmonary disease, *FF/VI* fluticasone furoate/vilanterol

Amongst new users of FF/VI who had at least two prescriptions and for which it was possible to observe a change in strength, changes from FF/VI 200/25 to FF/VI 100/25 (i.e., a reduction in strength) were more frequent in the COPD diagnosis group than an escalation from FF/VI 100/25 to the higher strength (26.2% and 4.5%, respectively), while in the asthma diagnosis group, reductions and escalations were equally likely (11.5% vs. 15.0%, respectively) (Fig. 2).

### Potential Off-Label Prescribing of FF/VI

Fewer than five children aged < 12 years received a prescription for FF/VI (all 100/25 formulation), representing less than 0.3% of the patients in the combined asthma and other diagnosis groups.

The upper-bound estimates of potential off-label prescribing of FF/VI 200/25 that assumed all COPD patients with an asthma history received FF/VI 200/25 off-label for treatment of COPD were 16.9% (448/2653) when considering only the index prescription and 20.2% (535/2653) when considering the 87 patients who escalated from FF/VI 100/25 to FF/VI 200/25 during the 12-month study period. The lower-bound estimates of potential off-label prescribing (which assumed all COPD patients with an asthma history received FF/VI 200/25 on-label for treatment of asthma) were 7.5% (198/2653) at the index date and 9.3% (246/2653) during the 12-month follow-up period.

## Discussion

Findings from this retrospective, longitudinal, observational study suggest that in the years immediately following the availability of FF/VI in the UK, most FF/VI initiators (77%) did so at the lower strength (100/25), and of these, the majority had a recorded diagnosis of COPD. The treatment pattern data for FF/VI indicated that most users did not have a change in FF/VI strength over a 12-month period, though this varied by diagnosis group and strength initiated.

Characteristics of new users of FF/VI 100/25, FF/VI 200/25, and other ICS/LABA FDC were largely similar within each diagnosis group in terms of demographics. Within the COPD diagnosis group, there was evidence of channelling of FF/VI to COPD patients with higher clinical severity, whereby new users of FF/VI, especially FF/VI 200/25, had a greater exacerbation burden and higher dyspnoea scores compared with patients who were prescribed another ICS/LABA FDC. Yet, FF/VI prescriptions were also recorded for patients with no exacerbation history recorded in primary care in the previous 12 months across the FF/VI 100/25, FF/VI 200/25, and other ICS/LABA FDC groups (36.5, 39.3, and 29.6% of patients, respectively, although it should be noted that the algorithm used to identify COPD exacerbations is only moderately sensitive, so these figures may overestimate the proportion of patients without an exacerbation in the prior 12 months [14]). Taken together, these observations demonstrate that ICS/LABA combinations are often correctly prescribed to patients at risk of COPD exacerbations in UK primary care [15], but may also be inappropriately prescribed to patients who do not experience exacerbations [16]. Of note, the patients experiencing exacerbations in this study who were prescribed FF/VI may also have derived more benefit from triple therapy than their prescribed combination per existing clinical evidence [17, 18]. These observations highlight a potential disconnect between guideline recommendations for COPD maintenance and true prescribing behaviours in UK primary care. Previous studies have also demonstrated similar issues in this setting, largely regarding the overprescription of ICS/LAMA/LABA triple therapy [19]. Inappropriate ICS prescription as described here and in previous studies exposes patients to unnecessary safety issues like pneumonia [20], and also increases the healthcare resource use associated with treatment [21].

FF/VI was very rarely prescribed to children aged < 12 years in the UK, which is consistent with the label. Few studies in the published literature have specifically examined ICS/LABA prescribing in children with asthma. In a Swedish register study, 15% of asthma medication prescriptions in children aged 0–17 were considered to be off-label [22]. In a study of prescribing in Europe, off-label prescribing of salbutamol in Dutch children < 18 months of age was 22.7 prescriptions per 100 person years, while off-label prescribing of fixed combinations of β_2_ mimetics plus anticholinergics in Dutch children > 6 years of age ranged from 0.5 to 3.5 prescriptions per 100 patient-years [23]. Whilst there are no studies to directly compare FF/VI potential off-label prescribing in children with other ICS/LABAs that also are not indicated in paediatric populations (Seretide 50/100 [salmeterol/fluticasone propionate] is licensed for children with asthma aged ≥ 4 years in the UK), the low level of off-label prescribing of FF/VI in children under 12 observed suggests that physicians are aware that this new medicine is not licensed for paediatric prescribing.

Based on the upper estimate, as many as one in five new users of FF/VI with COPD in the UK were prescribed FF/VI 200/25, potentially off-label. Defining COPD off-label prescribing in this retrospective database study was challenging because a large proportion of the COPD patients met the definition of having an asthma history, and the specific disease indication associated with each prescription of FF/VI is not routinely recorded in electronic medical records. To overcome this challenge, we chose to present a range of off-label prescribing estimates representing the extremes of two scenarios where either all or none of the COPD patients with an asthma history were prescribed FF/VI 200/25 for their asthma. Another alternative approach would have been to estimate possible off-label prescribing only in the subgroup of COPD patients without any evidence of historical asthma. Indeed, this approach results in off-label estimates of 14.1% on the index date and 17.5% at any time during follow-up, which are within the range described when using the full COPD population.

In exploring these one in five potentially off-label patients in more detail, we observed that many had received a high-dose ICS in the past, and over half were using a LAMA concomitantly (triple therapy), which is recommended for more severe patients [15]. In addition, these COPD patients also experienced a greater exacerbation burden. These observations may suggest that some clinicians attempt to control more severe COPD with a high dose of steroid; however, it is not possible to determine physicians’ motivation from electronic medical records alone. We also noted that not all COPD patients who initiated FF/VI 200/25 continued to receive prescriptions of FF/VI 200/25 for the duration of follow-up; just over a quarter received only one FF/VI 200/25 prescription during follow-up, and another 26% were eventually changed to the lower FF/VI strength. In comparison, only a small proportion of FF/VI 100/25 initiators with COPD shifted to the higher strength during the 12-month study period. Our data also indicated that patients’ prescriptions covered a median of 7 (FF/VI 100/25) and 5 (FF/VI 200/25) months of the study follow-up period. While it is possible that some physicians may have prescribed ICS/LABA only for acute episodes and not for maintenance during this study, we did not assess prescribing in relation to the timing of exacerbations. Alternative explanations for the low median prescription coverage may include: treatment switching and discontinuation, as noted above; loss to follow-up, as approximately a quarter of patients did not contribute a full 12 months of follow-up data, commonly as a result of switching GP practice during this period; prescribing as a therapeutic trial, whereby physicians were cautious in providing lengthy prescriptions for a novel treatment and low adherence. Among patients for whom the full 12 months of follow-up data were available, however, 44% had a medication possession ratio of at least 0.8, and 59% had a proportion of days covered by their prescriptions of at least 0.8, indicating a relatively high level of adherence to the prescribed treatment. Comparison studies of off-label medication use in COPD are not yet available. Within this study, it was not possible to directly compare the potential off-label prescribing of FF/VI with the other ICS/LABA FDC cohort; the other ICS/LABA group comprised products with varying doses of ICS, indications, and age groups, making the calculation of class level off-label prescribing inappropriate.

A strength of the study was the use of CPRD data containing rich and detailed longitudinal clinical records for a large population-based sample of patients treated for asthma and COPD in UK primary care. CPRD data have been widely used for observational research, including drug utilisation studies of prescription medications in respiratory diseases [24], and validation studies of physician-recorded diagnosis of COPD [11] and asthma [12]. While broadly representative of the UK population in terms of age, sex, and ethnicity [10], CPRD data is not a random sample. Nevertheless, the findings are likely to be applicable to other new users of FF/VI in the UK, though they may not reflect patterns in other countries.

There are several limitations inherent with the study design that should be considered when interpreting the findings. Firstly, GPs in the UK are not required to record the indication for every prescription they issue, and thus in this study we were unable to state with absolute certainty why a GP prescribed FF/VI or another ICS/LABA FDC to their patients. Secondly, there is the potential for misclassification of patients into the diagnosis groups. For example, the other diagnosis group could have included patients with COPD that did not meet the age requirement, or patients with COPD and/or asthma without a coded diagnosis in their available medical record (or only available in free text). These un- (or under)recorded COPD or asthma patients could have affected the quantification of potential off-label use. As less than 4% of new users of FF/VI had neither a COPD or asthma diagnosis, we expect any impact on the results to be minimal. We attempted to limit misclassification by using published validated diagnosis codes for asthma [12] and COPD [11]. This classification could have potentially been improved if spirometry criteria were included in the COPD diagnosis group definition; however, we observed that 73–80% of patients in the COPD group did have an FEV_1_/FVC ratio < 70%, suggesting airflow limitation in line with a COPD diagnosis.

## Conclusion

This study suggests that the prescribing of FF/VI is rare in children under 12 years of age in the UK. Among adults, up to one in five COPD patients in the UK have been prescribed the higher strength FF/VI 200/25 formulation. Some of this potential off-label prescribing may be linked to historical or concurrent asthma, and/or channelled to patients with more severe COPD and prior treatment with high-dose steroids. While the demographic characteristics of patients newly initiating FF/VI and other ICS/LABA FDC were quite similar, there was some evidence of the channelling of FF/VI to more severe COPD patients.

## Electronic supplementary material

Below is the link to the electronic supplementary material.

Supplementary material 1 (DOCX 261 kb)
