# Supplementary material for: Treatment Patterns of New Users of Fluticasone Furoate/Vilanterol in Asthma and COPD in UK Primary Care: Retrospective Cohort Study
Source: Pulm Ther. 2019 Apr 24;5(1):81–95. doi: 10.1007/s41030-019-0092-z (PMC6967316; doi:10.1007/s41030-019-0092-z)
Supplement: Supplementary file 1 — Supplementary material 1 (DOCX 261 kb) [file 41030_2019_92_MOESM1_ESM.docx]

Supplemental Appendix

Table S1 Demographic characteristics at baseline for COPD diagnosis group, with and without a history of asthma, by index medication

|  | | **Patients in the COPD diagnosis group with a history of asthma (N=7,343)^a^** | | | | | | **Patients in the COPD diagnosis group without a history of asthma (N=9,316)^a^** | | | | | |
| --- | --- | --- | --- | --- | --- | --- | --- | --- | --- | --- | --- | --- | --- |
|  |  | FF/VI 100/25 | | FF/VI 200/25 | | Other ICS/LABA FDC | | FF/VI 100/25 | | FF/VI 200/25 | | Other ICS/LABA FDC | |
|  |  | *n* = 994 | | *n* = 250 | | *n* = 6,769 | | *n* = 1,211 | | *n* = 198 | | *n* = 8,807 | |
|  |  | No. | (%)^b^ | No. | (%)^b^ | No. | (%)^b^ | No. | (%)^b^ | No. | (%)^b^ | No. | (%)^b^ |
| **Age at index date** | |  |  |  |  |  |  |  |  |  |  |  |  |
|  | Mean (SD) | 68.5 | 10.7 | 66.8 | 11.7 | 68.4 | 11.7 | 70.1 | 10.1 | 69.7 | 10.1 | 69.4 | 10.8 |
| **Gender** | |  |  |  |  |  |  |  |  |  |  |  |  |
|  | Female | 556 | 55.9 | 139 | 55.6 | 3,790 | 56.0 | 549 | 45.3 | 70 | 35.4 | 4,065 | 46.2 |
| **Smoking status** | |  |  |  |  |  |  |  |  |  |  |  |  |
|  | Current smoker | 355 | 35.7 | 82 | 32.8 | 2,322 | 34.3 | 486 | 40.2 | 79 | 39.9 | 3,809 | 43.4 |
|  | Ex-smoker | 530 | 53.3 | 124 | 49.6 | 3,349 | 49.5 | 670 | 55.4 | 107 | 54.0 | 4,310 | 49.1 |
|  | Never smoker | 109 | 11.0 | 44 | 17.6 | 1,096 | 16.2 | 54 | 4.5 | 12 | 6.1 | 662 | 7.5 |
|  | *Missing^c^* | *0* | *0.00* | *0* | *0.00* | *2* | *0.03* | *1* | *0.1* | *0* | *0.00* | *26* | *0.3* |
| **Body mass index (kg/m^2^)** | |  |  |  |  |  |  |  |  |  |  |  |  |
|  | Mean (SD) | 28.7 | 6.4 | 29.2 | 7.0 | 28.3 | 6.6 | 27.2 | 6.2 | 27.1 | 6.7 | 27.1 | 6.4 |
|  | *Missing^c^* | *40* | *4.0* | *8* | *3.2* | *422* | *6.2* | *46* | *3.8* | *17* | *8.6* | *736* | *8.4* |
| **Mean (SD) follow up time (days)** | | 329 (85) | | 329 (85) | | 321 (93) | | 327 (87) | | 317 (101) | | 317 (101) | |

*COPD* chronic obstructive pulmonary disease, *FF/VI* fluticasone furoate/vilanterol, *ICS/LABA* inhaled corticosteroid/long-acting beta agonist, *SD* standard deviation

^a^Patients can qualify for more than one qualifying index medication which is reflected in the higher number of records when summing across the exposure cohorts

^b^Unless otherwise specified

^c^Percentages were calculated separately for those with missing and without missing data

Fig. S1 Frequency of medication prescribed to patients in the COPD diagnosis group within the 12 months prior to the index date


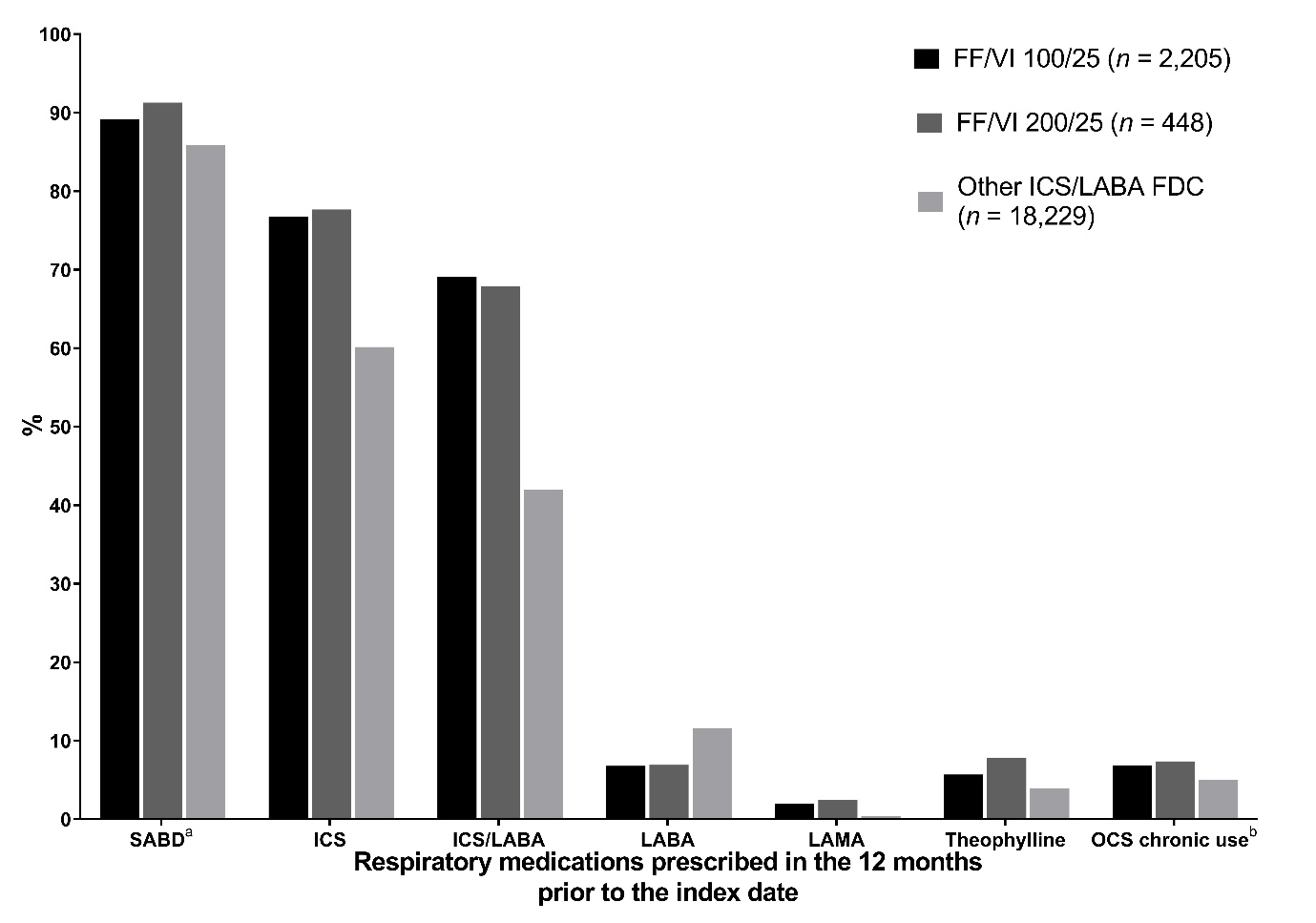


*FDC* fixed dose combination, *FF/VI* fluticasone furoate/vilanterol, *ICS* inhaled corticosteroids, *LABA* long-acting beta-agonist, *LAMA* long-acting muscarinic antagonists, *OCS* oral corticosteroids, *SABA* short-acting beta agonists, *SABD* short-acting bronchodilators, *SAMA* short-acting muscarinic antagonist

Only FDC of ICS/LABA and LABA/LAMA are considered

^a^Includes the following COPD 'reliever' medications: SABA, SAMA, fixed combinations of SABA and cromoglycate, and fixed combinations of SABA and SAMA

^b^Defined as at least four prescription records with a maximum gap between two prescriptions equal to 30 days

Fig. S2 Frequency of medication prescribed to patients in the Asthma diagnosis group within

the 12 months prior to the index date


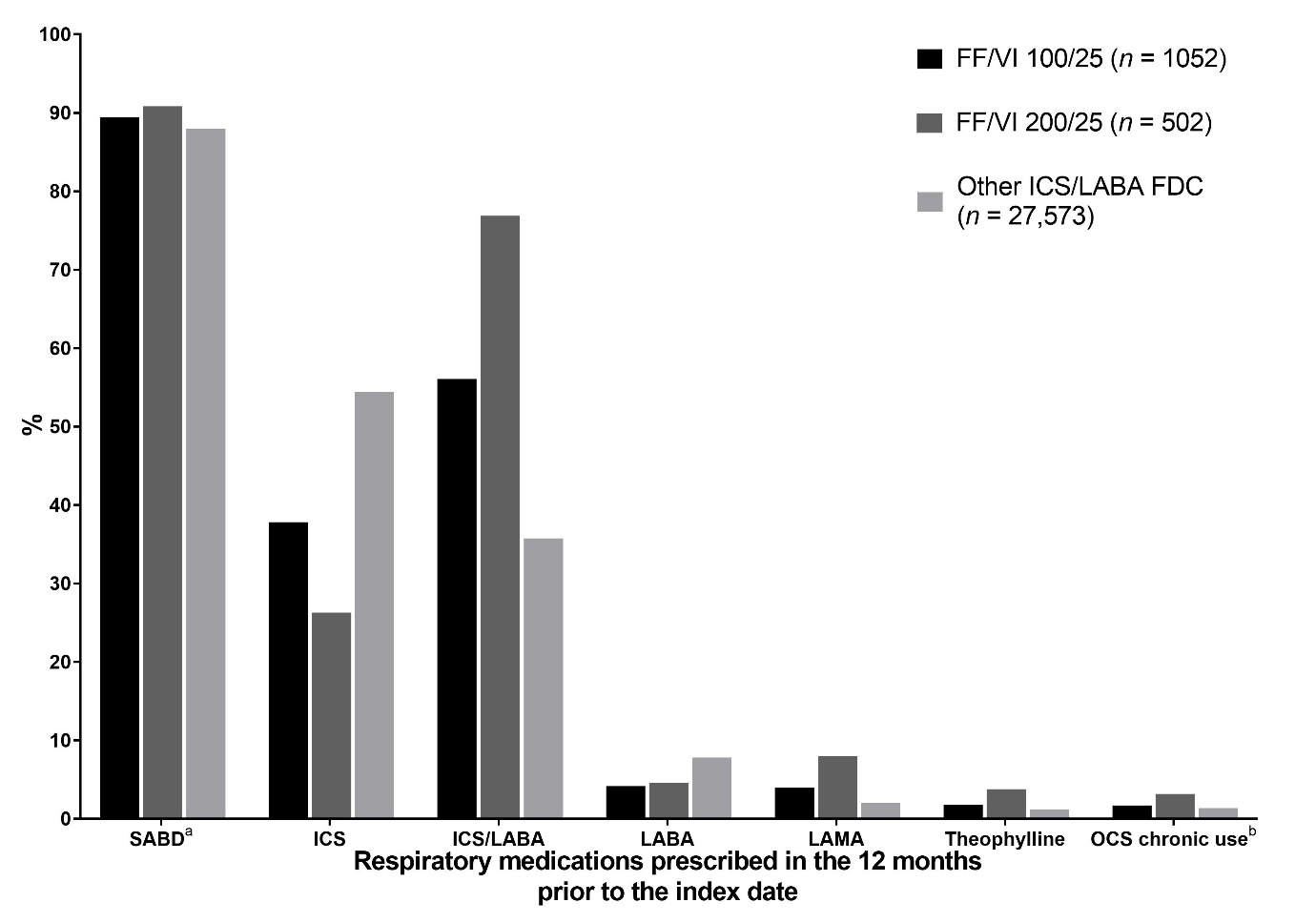


*FDC* fixed dose combination, *FF/VI* fluticasone furoate/vilanterol, *ICS* inhaled corticosteroids, *LABA* long-acting beta-agonist, *LAMA* long-acting muscarinic antagonists, *OCS* oral corticosteroids, *SABA* short-acting beta agonists, *SABD* short-acting bronchodilators, *SAMA* short-acting muscarinic antagonist

Only FDC of ICS/LABA are considered

^a^Includes the following asthma 'reliever' medications: SABA, SAMA, fixed combinations of SABA and cromoglycate, and fixed combinations of SABA and SAMA

^b^Defined as at least four prescription records with a maximum gap between two prescriptions equal to 30

**Table S2** Disease severity at baseline for the COPD diagnosis group, Asthma diagnosis group and Other diagnosis groups by index medication in the HES-linked sample

| Patients in the COPD diagnosis group | | | | | | | |
| --- | --- | --- | --- | --- | --- | --- | --- |
|  | | FF/VI 100/25  N=1,347 | | FF/VI 200/25  N=378 | | Other ICS/LABA FDC  N=24,280 | |
|  |  | No. | (%)^a^ | No. | (%)^a^ | No. | (%)^a^ |
| COPD exacerbations at baseline (recorded in primary and/or secondary care) | Rate per person year (95% CI) | 1.43 | (1.35, 1.51) | 1.58 | (1.41, 1.77) | 1.26 | (1.21, 1.26) |
|  | 0 events | 334 | 37.11 | 61 | 32.11 | 3,120 | 39.89 |
|  | 1 event | 232 | 24.78 | 52 | 27.37 | 2,186 | 27.95 |
|  | ≥ 2 events | 334 | 37.11 | 77 | 40.53 | 2,516 | 32.17 |
| FEV_1_ percent predicted at baseline | Mean (SD) | 57.70 | 19.44 | 55.20 | 17.92 | 57.17 | 18.95 |
|  | Mild, Grade 1 (≥ 80%) | 91 | 12.01 | 16 | 10.06 | 738 | 11.91 |
|  | Moderate, Grade 2 (≥ 50% to < 80%) | 396 | 52.24 | 81 | 50.94 | 3,142 | 50.72 |
|  | Severe, Grade 3 (≥ 30% to < 50%) | 221 | 29.16 | 51 | 32.08 | 1,909 | 30.82 |
|  | Very severe, Grade 4 (< 30%) | 50 | 6.60 | 11 | 6.92 | 406 | 6.55 |
|  | *Missing^b^* | *142* | *15.78* | *31* | *16.32* | *1,627* | *20.80* |
| FEV_1_/FVC ratio at baseline | Mean (SD) | 60.54 | 18.97 | 60.69 | 20.43 | 61.10 | 16.09 |
|  | < 70% | 541 | 78.86 | 101 | 71.13 | 4,059 | 72.75 |
|  | *Missing^b^* | *214* | *23.78* | *48* | *25.26* | *2,243* | *28.68* |
| Dyspnoea (MRC grade) at baseline | Mean (SD) | 2.82 | 0.99 | 2.80 | 1.10 | 2.7 | 1.00 |
|  | Grade 1 | 58 | 7.65 | 15 | 10.14 | 563 | 10.02 |
|  | Grade 2 | 250 | 32.98 | 51 | 34.46 | 2,010 | 35.78 |
|  | Grade 3 | 252 | 33.25 | 40 | 27.03 | 1,784 | 31.76 |
|  | Grade 4 | 167 | 22.03 | 32 | 21.62 | 1,065 | 18.96 |
|  | Grade 5 | 31 | 4.09 | 10 | 6.76 | 195 | 3.47 |
|  | *Missing^b^* | *142* | *15.78* | *42* | *22.11* | *2,205* | *28.19* |
| Patients in the Asthma diagnosis group | | | | | | | |
|  |  | FF/VI 100/25  N=404 | | FF/VI 200/25  N=170 | | Other ICS/LABA FDC  N=13,708 | |
|  |  | No. | (%)^a^ | No. | (%)^a^ | No. | (%)^a^ |
| Asthma exacerbation at baseline (recorded in primary and/or secondary care) | Rate per person year (95% CI) | 0.13 | (0.10 8, 0.17) | 0.27 | (0.20, 0.36) | 0.13 | (0.12, 0.13) |
|  | 0 events | 362 | 89.60 | 142 | 83.53 | 12,254 | 89.39 |
|  | 1 event | 33 | 8.17 | 21 | 12.35 | 1,263 | 9.21 |
|  | ≥ 2 events | 9 | 2.23 | 7 | 4.12 | 191 | 1.39 |

*CI* confidence interval, *COPD* chronic obstructive pulmonary disease, *FDC* fixed-dose combination, *FEV_1_* forced expiratory volume in 1 second, *FF/VI* fluticasone furoate/vilanterol, *FVC* forced vital capacity, *ICS/LABA* inhaled corticosteroid/long-acting beta agonist, *MRC* Medical Research Council, *SD* standard deviation

^a^Unless otherwise specified

^b^Percentages were calculated separately for those with missing and without missing data

**Table S3** Adherence to FF/VI therapy in the first 12 months following initiation, for patients with ≥12 months of follow-up data

| All patients initiating FF/VI with 12 months follow up (N=3,312) | | | | | | | |
| --- | --- | --- | --- | --- | --- | --- | --- |
|  | | COPD diagnosis group | | Asthma diagnosis group | | Other diagnosis group | |
|  |  | No.^a^ | (%)^a^ | No.^a^ | (%)^a^ | No.^a^ | (%)^a^ |
| Medication possession ratio | Total patients^b^ | 1,815 | 63 | 987 | 34 | 72 | 3 |
|  | Mean (SD) | 0.87 | 0.42 | 0.84 | 0.99 | 0.82 | 0.33 |
|  | Median (IQR)^c^ | 0.92 | 0.63 -1.05 | 0.82 | 0.58 - 1.01 | 0.86 | 0.60 -1.00 |
|  | Min, Max | 0.09, 10.00 | | 0.08, 30.00 | | 0.12, 2.14 | |
|  | <80% | 690 | 38.02 | 485 | 49.14 | 28 | 38.89 |
|  | ≥80% | 1,125 | 61.98 | 502 | 50.86 | 44 | 61.11 |
| Proportion of days covered | Total patients | 2,042 | 61.65 | 1,167 | 35.24 | 103 | 3.11 |
|  | Mean (SD) | 0.66 | 0.33 | 0.59 | 0.34 | 0.42 | 0.35 |
|  | Median (IQR) | 0.74 | 0.41 - 0.99 | 0.66 | 0.25 - 0.90 | 0.25 | 0.08 - 0.74 |
|  | Min, Max | 0.08, 1.00 | | 0.08, 1.00 | | 0.08, 1.00 | |
|  | <80% | 1,087 | 53.23 | 727 | 62.30 | 79 | 76.70 |
|  | ≥80% | 955 | 46.77 | 440 | 37.70 | 24 | 23.30 |

*COPD* chronic obstructive pulmonary disease, *FF/VI* fluticasone furoate/vilanterol, *IQR* interquartile range, *SD* standard deviation

^a^Unless otherwise specified

^b^The medication possession ratio was not calculated in 438 patients who only received one prescription for FF/VI

^c^927 patients were in possession of FF/VI for greater than 365 days (hence some upper IQR and Max values are greater than 1.00)

***Hospital Episode Statistics (HES)-linked subset sample.***

A subset of patients from the CPRD full sample were linked to the Hospital Episode Statistics (HES) data held by CPRD to obtain additional patient-level data on secondary care. Linkage of CPRD-GOLD data to other patient level datasets such as Hospital Episodes Statistics (HES) currently includes patients from 407 practices. These linkages cover approximately 75% of contributing CPRD GOLD practices in England, and roughly 57% of contributing CPRD GOLD practices in the UK.

HES data were used in addition to GP data to capture COPD and asthma exacerbations treated in the secondary, hospitalised setting which may, or may not, be reported back to the GP or recorded in the GP database. Acute exacerbations of COPD (AECOPD) in the 12 months prior to the index date were defined using a validated algorithm [1].

The linked HES cohort comprised 1,725 patients taking FF/VI and 24,280 taking other ICS/LABA (Fig. S1). Asthma and COPD disease severity in the HES linked cohort is shown in Table S2.

**REFERENCE**

1. Rothnie KJ, Müllerová H, Hurst JR, et al. Validation of the Recording of Acute Exacerbations of COPD in UK Primary Care Electronic Healthcare Records. PLoS One*.* 2016*;*11:e0151357.
